# Neurological Effects of Repeated Blast Exposure in Special Operations Personnel

**DOI:** 10.1089/neu.2023.0309

**Published:** 2024-04-04

**Authors:** James R. Stone, Brian B. Avants, Nicholas J. Tustison, Jessica Gill, Elisabeth A. Wilde, Kiel D. Neumann, Leslie A. Gladney, Madison O. Kilgore, F. Bowling, Christopher M. Wilson, John F. Detro, Heather G. Belanger, Katryna Deary, Hans Linsenbardt, Stephen T. Ahlers

**Affiliations:** ^1^ Department of Radiology and Medical Imaging, University of Virginia, Charlottesville, Virginia, USA.; ^2^ School of Nursing, Johns Hopkins University, Baltimore, Maryland, USA.; ^3^ Department of Neurology, University of Utah, Salt Lake City, Utah, USA.; ^4^ George E. Wahlen VA, Salt Lake City Health Healthcare System, Salt Lake City, Utah, USA.; ^5^ Molecular Imaging Research Hub, St. Jude Children's Research Hospital, Memphis, Tennessee, USA.; ^6^ U.S. Special Operations Command, Tampa, Florida, USA.; ^7^ Departments of Psychiatry and Behavioral Neurosciences, and Psychology, University of South Florida, Tampa, Florida, USA.; ^8^ Cognitive Research Corporation, St. Petersburg, Florida, USA.; ^9^ Naval Medical Research Command, Silver Spring, Maryland, USA.; ^10^ Operational and Undersea Medicine Directorate, Naval Medical Research Command, Silver Spring, Maryland, USA.

**Keywords:** military, neuroinflammation, neuroimaging, repeated blast exposure, serum biomarker

## Abstract

Exposure to blast overpressure has been a pervasive feature of combat-related injuries. Studies exploring the neurological correlates of repeated low-level blast exposure in career “breachers” demonstrated higher levels of tumor necrosis factor alpha (TNFα) and interleukin (IL)-6 and decreases in IL-10 within brain-derived extracellular vesicles (BDEVs). The current pilot study was initiated in partnership with the U.S. Special Operations Command (USSOCOM) to explore whether neuroinflammation is seen within special operators with prior blast exposure. Data were analyzed from 18 service members (SMs), inclusive of 9 blast-exposed special operators with an extensive career history of repeated blast exposures and 9 controls matched by age and duration of service. Neuroinflammation was assessed utilizing positron emission tomography (PET) imaging with [^18^F]DPA-714. Serum was acquired to assess inflammatory biomarkers within whole serum and BDEVs. The Blast Exposure Threshold Survey (BETS) was acquired to determine blast history. Both self-report and neurocognitive measures were acquired to assess cognition. Similarity-driven Multi-view Linear Reconstruction (SiMLR) was used for joint analysis of acquired data. Analysis of BDEVs indicated significant positive associations with a generalized blast exposure value (GBEV) derived from the BETS. SiMLR-based analyses of neuroimaging demonstrated exposure-related relationships between GBEV, PET-neuroinflammation, cortical thickness, and volume loss within special operators. Affected brain networks included regions associated with memory retrieval and executive functioning, as well as visual and heteromodal processing. Post hoc assessments of cognitive measures failed to demonstrate significant associations with GBEV. This emerging evidence suggests neuroinflammation may be a key feature of the brain response to blast exposure over a career in operational personnel. The common thread of neuroinflammation observed in blast-exposed populations requires further study.

## Introduction

Exposure to blast overpressure has been a pervasive feature of combat-related injuries experienced by operational personnel. In addition to combat-related blast-induced traumatic brain injury (b-TBI), concern exists that repeated exposure to low-intensity blasts during day-to-day training and operations may be linked to long-term neurological sequelae. This concern has fueled research exploring potential cumulative injury mechanisms following repeated blast exposure. A major focus of these efforts has been to assess the effects of repeated low-intensity blast exposure in military operational personnel with repeated exposures throughout their military careers. Low-level blast has been previously described as referring to overpressure generated by outgoing munitions, which tends to be lower in intensity as compared with high-level blast, which has been described as overpressure generated by incoming munitions.^1^ A number of studies have explored neurological sequelae in “breachers,” who employ explosives to breach hardened structures, such as walls or buildings.

Breachers are routinely exposed to repeated low-level blasts during training and operations.^2^ However, exposure to these individual low-level blasts is not associated with an injury-level event. A study examining anonymous survey respondents from military and non-military law enforcement populations reported an increased prevalence and severity of symptoms in chronically blast-exposed populations, including descriptions of headaches, difficulty remembering, slowed thinking, loss of energy, feelings of aggression, social withdrawal, and irritability.^3^ This work suggests subclinical effects associated with repeated low-level blast exposure may accumulate over time and culminate in clinically significant chronic symptoms. More broadly, epidemiological studies examining the relationship between military occupational specialties (MOS) associated with high risk of blast exposure and neurologically related medical outcomes have demonstrated increased prevalence of neurosensory abnormalities, TBI, cognitive abnormalities, headaches, behavioral health conditions, substance abuse, and symptoms such as anxiety, fatigue, and migraines in high-risk MOS as compared with other specialties.^4,5^ Additionally, high-risk MOS were more likely to undergo evaluation for disability or be medically separated from the military.^4,5^

A study performed by our group that assessed experienced breachers with a career history of repeated blast exposure demonstrated several significant alterations in blast-exposed populations compared with controls. These included symptoms of tinnitus, light and noise sensitivity, irritability, and memory alterations along with postural instability and hearing loss.^6,7^ Additionally, magnetic resonance imaging (MRI) demonstrated alterations in brain structure and function, including alterations in cortical thickness, decreased white matter integrity, and reductions in activation of the default mode network (DMN).^8^ Among the findings within experienced breachers were elevated markers of neuroinflammation within brain-derived extracellular vesicles (BDEVs) isolated from peripheral blood. Specifically, increases in pro-inflammatory tumor necrosis factor alpha (TNFα) and interleukin (IL)-6 and decreases in anti-inflammatory IL-10 were noted, resulting in an elevation of the IL-6/IL-10 ratios, consistent with increased inflammation in the brains of experienced breachers.^9^ Additionally, a pilot study of whole-blood transcriptomic analysis demonstrated a number of dysregulated genes related to chronic inflammation and immune response within experienced breachers compared with well matched controls.^10^

The finding of elevated neuroinflammation within blast-exposed populations may have implications for the long-term health of blast-exposed service members (SMs). Neuroinflammation plays a critical role in the immune response following injury, where microglia and astrocytes play a key role in mediating the overall neuroinflammatory response.^11,12^ Although the neuroinflammatory response is potentially adaptive in early recovery intervals, there is growing evidence that injury may also initiate a prolonged and maladaptive pro-inflammatory response that can result in deleterious changes to brain structure^12,13^ and function.^14^ Many neurodegenerative conditions, such as Alzheimer's disease (AD), Parkinson's disease (PD), and amyotrophic lateral sclerosis (ALS), share a common theme of chronic neuroinflammation^15^ that may be detectable in prodromal periods, and that may also share links to TBI.^16^ Accumulating evidence suggests that dysregulated neuroinflammation can persist for years or decades following TBI, leading to secondary tissue compromise, and this has been associated with poorer outcome.^17^

Recent human studies have employed positron emission tomography (PET) radiopharmaceuticals to image neuroinflammation. One of the more widely used class of radiopharmaceuticals in the study of the neuroinflammatory response targets the translocator protein (TSPO). TSPO is an 18 kDa mitochondrial associated protein with a variety of functions throughout the body including cholesterol transport, bile acid synthesis, cardiac contractility, apoptosis, stress adaptation, and modulation of the immune system. Within the brain, TSPO is found within microglia and demonstrates significantly increased expression within activated microglia. As such, radiopharmaceuticals consisting of a TSPO ligand coupled to a PET radioisotope serve as effective imaging agents for neuroinflammation.

A study of former athletes demonstrated significant increase in uptake of the TSPO ligand [^11^C]DPA-713 within former National Football League (NFL) players as compared with healthy, age-matched controls.^18^ Similarly, the TSPO ligand [^18^F]DPA-714 was recently used to study microglial activation in collegiate athletes following a sports concussion, compared with healthy, age-matched controls.^19^ That study demonstrated persistent elevated neuroinflammation in collegiate athletes who were diagnosed with a sport concussion and cleared for unrestricted return to play based on a clinical recovery. Using the TSPO ligand [^11^C]PK11195, a recent study showed increased neuroinflammation in the hippocampus of athletes with sports-related concussion.^20^ Additionally, [^11^C]PK11195 has been used to demonstrate elevated neuroinflammation 6 months following injury in patients with TBI as compared with age-matched healthy controls.^21^

Based on the above, a pilot study was initiated by our group in partnership with the U.S. Special Operations Command (USSOCOM) to explore whether neuroinflammation is seen within special operators with a history of prior blast exposure. This study was designed as an initial feasibility effort to recruit a small number of Special Operations Forces (SOF) SMs with exposure to repeated low-level blasts over their careers. The study is the first to explore neuroinflammation as a pivotal mechanism through which blast-related changes impact brain structure and function. Further, this is the first work to explore neuroimaging-derived critical generalized blast exposure value (GBEV) thresholds for blast exposure.

## Methods

All procedures were reviewed and approved by the Institutional Review Board at the University of Virginia (UVA) and the study protocol was approved by the Naval Medical Research Center Institutional Review Board in compliance with all applicable federal regulations governing the protection of human subjects. The investigators have adhered to the policies for protection of human subjects as prescribed in AR 70-25.

Informed consent was provided by all participants and procedures were performed during a 1-day evaluation at UVA. A total of 21 SMs were recruited into experimental and control groups for the study. SMs were screened for inclusion in the study by a SOCOM nurse practitioner located at MacDill Air Force Base in Tampa, Florida. SMs within the experimental group must have had a prior history of blast exposure in training or operations. Subjects in the control group must not have had a history of previous exposure to explosives, including but not limited to explosive entry (breacher) operations/training, heavy weapons use, and/or explosives ordinance disposal (EOD). A history of moderate or severe TBI as defined by the American Congress of Rehabilitation, MRI contraindications, current severe medical condition, current diagnosis of central nervous system (CNS) disorder other than mild TBI, and any cardiac, respiratory, or other medical condition that may affect cerebral metabolism were all criteria for exclusion from the study.

### Demographics, clinical history, and neuropsychological assessment

Participant history was acquired, including demographics, military history, and medical history, along with a head injury questionnaire. The head injury questionnaire included eliciting a history of cause of injury, age of injury, loss of consciousness, presence of amnesia related to the event, changes in mood, sleep problems, or other associated clinical symptoms. Additionally, subjects were administered the Naval Medical Research Center (NMRC) Blast Exposure Threshold Survey (BETS).^22^ The BETS elicits information on exposure to weapons and explosives, injury history, auditory symptoms, vestibular symptoms, mood issues, sleep issues, and cognitive symptoms. Neuropsychological testing and administered inventories included the Automated Neuropsychological Assessment Metrics 4 TBI-MIL (ANAM4 TBI-MIL), Combat Exposure Scale (CES), Pittsburgh Sleep Quality Index (PSQI), PTSD Checklist for DSM-5 (PCL-5), Psychological General Well-Being Index (PGWI), and Neurobehavioral Symptom Inventory (NSI).

### Generalized blast exposure value (GBEV)

The BETS with the accompanying GBEV was recently developed specifically for military SMs to relate the type and amount of blast exposure to reported symptomology.^22^ This value is analogous to the Cumulative Head Impact Index (CHII) for former high school and college football players.^23^ The motivation for such measures is to simultaneously characterize a specific population and identify brain trauma exposure, relate that exposure to outcomes, and determine a threshold for increased risk of long-term problematic health outcomes. Analogously, the GBEV is determined by the following formula:

$$\begin{array}{cc} GBEV & =0.976\left(1BEC\right)+288\left(2BEC\right)+41\left(3BEC\right)\\ & +77\left(4BEC\right)\left(4freq\right)+75\left(5BEC\right)\left(5freq\right) \end{array}$$

where *BEC* is the “blast exposure count” defined as the product of 1) years of experience with a weapon, 2) months of experience per year, 3) days of experience per month, and 4) number of exposures per day. Categorization of Light arms, Artillery, Recoilless rifles, and Explosives (CLARE) are denoted for each term in parentheses: (1BEC) small arms; (2BEC) large arms (including shoulder-fired); (3BEC) artillery (or large weapons carried by a vehicle); (4BEC) small explosives; and (5BEC) large explosives. *freq* refers to the daily frequency variable for each category.

### Serum biomarkers

All samples were measured in a fully blinded manner. Blood samples were collected into tubes with ethylenediaminetetraacetic acid (EDTA) and centrifuged before being aliquoted and placed in the freezer at −80°C for long-term storage. Extracellular vesicles (EVs) were isolated from thawed samples as previously published.^24^ Of thawed samples, 500 μL were defibrinated with thromboplastin D and incubated at room temperature for 30 min before centrifuging for 5 min at 10,000*g* at 4°C. The supernatant was transferred into fresh tubes with added ExoQuick exosome solution, mixed, and incubated for 60 min at 4°C before centrifuging. The resulting pellet containing all EVs was resuspended in 500 μL ultra-pure water and placed at −80°C for long-term storage. EVs were precipitated by using ExoQuick plasma prep and exosome precipitation kits (System Bioscience, Inc.). Five microliters of thrombin (5000 U/mL in phosphate-buffered saline [PBS]) were added to 500 μL plasma to a final concentration of 5 U/mL thrombin. Tubes were incubated at room temperature (25°C) for 5 min while gently mixing prior to centrifugation at 10,000 rpm for 5 min. The resulting supernatant was transferred to a new tube with ExoQuick to precipitate EVs for 30–60 min at 4°C. Then, vials were centrifuged at 1500*g* for 30 min. The resulting EV pellet at the bottom of the tube was resuspended in 500 μL of PBS.

BDEVs were enriched in aqueous solution by precipitating with neuronal-specific antibodies SNAP25, PSA-NCAM, and CD171 from total circulating EVs from the plasma. Exosomal and plasma levels of tau, neurofilament light chain (NfL), and glial fibrillary acidic protein (GFAP) were analyzed using a high-sensitivity Simoa HD-X analyzer (Quanterix, Lexington, MA, USA) and a paramagnetic bead-based enzyme-linked immunosorbent assay (ELISA) according to the manufacturer's protocol. Coefficient of variation (intra- and inter-plate) values were below 15% for all analytes. Coreplex cytokines panel of IL-6, IL-10, and TNFα were analyzed using an SP-X Imaging and Analysis System (Quanterix). Because the array volumes are approximately 2 billion times smaller than an ELISA, a rapid buildup of fluorescent product is generated if labeled protein is present, and it provides detection ability between 100 and 1000 times that of ELISA methods. With diffusion defeated, this high local concentration of product can be readily observed. Samples were distributed randomly across plates and all assays were run in duplicate.

### Radiotracer synthesis

[^18^F]DPA-714 was synthesized in accordance with U.S. Pharmacopeia (USP)<823> guidelines by the University of Virginia Radiochemistry Core, as previously described.^25^ [^18^F]DPA-714 was synthesized in a 13.5 ± 3.04% radiochemical yield (EOB) with a molar activity of 34.3 ± 9.9 Ci/μmol (1258 ± 366 GBq/μol; *n* = 3) in a total synthesis time of 90 min. In all cases, radiochemical purity was >99%.

### Neuroimaging

#### PET-CT

Imaging was performed with a Siemens Biograph mCT (PET-CT) scanner (Siemens Healthineers, Knoxville, TN, USA). Measurements of body height and weight were performed before scan acquisition. A computed tomography (CT) scan was performed with a 100 mAs tube current, 100 kV voltage, 64 × 0.6 mm collimation, pitch of 0.55, reconstruction at a 2-mm slice thickness, and scanning time of 17.3 sec. Subjects received an intravenous, bolus injection of [^18^F]DPA-714 (296 ± 17 MBq) at the onset of a 90-min dynamic list mode PET acquisition. Genotyping was performed to determine TSPO polymorphism status for each subject. PET image reconstruction was performed at an image size of 400 with a 2.0 amplification factor and Gaussian filter with a full-width half maximum of 2.0. The TrueX + time of flight (TOF) method was used with five iterations and 21 subsets. Attenuation correction was performed using the NeuroAC calculated attenuation correction method.^26^ Using MiM version 6.9.7, (MiM Software, Inc., Cleveland, OH, USA), reconstructed and attenuation corrected images underwent motion correction, and static images binned at 20–90 min were generated using SumIP.

#### MRI

MRI neuroimaging was employed and the protocol included three-dimensional (3D) T1-weighted imaging (isotropic 1.0-mm spatial resolution), diffusion tensor imaging (DTI; (isotropic 1.5-mm spatial resolution, multi-shell with b-values of 1500 and 3000 s/mm^2^; 98 directions), resting state blood oxygen dependent (BOLD) imaging (10-min resting state acquisition, isotropic 2-mm spatial resolution, and 800-msec temporal resolution), susceptibility weighted imaging (3D gradient echo), and 3D arterial spin labeling (ASL; gradient-and spin-echo [GRASE] acquisition module and pseudo-continuous blood tagging). In addition, calibration/correction data were acquired to characterize spatial distortions in echo-planar-based acquisitions (BOLD and diffusion) and static field inhomogeneity. All data were collected on a state-of-the-art 3 Tesla MR scanner (Prisma, Siemens Healthineers, Erlangen, Germany) using a 64-channel head radiofrequency coil. The total acquisition time was roughly 1.5 h.

### Data processing

**FIG. 1.**
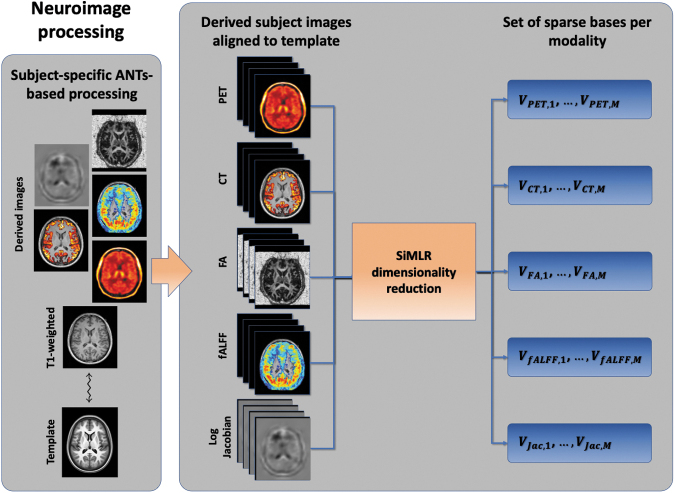
Neuroimage processing diagram for this study. Following image acquisition, computational processing generates several derived images per subject, which are registered to the study template via each subject's T1-weighted MRI. These aligned images are organized per modality, which are reduced in terms of dimensionality to representative bases using the SiMLR framework. CT, computed tomography; FA, fractional anisotropy; fALFF, fractional amplitude of low-frequency fluctuation; MRI, magnetic resonance imaging; PET, positron emission tomography; SiMLR, Similarity-driven Multi-view Linear Reconstruction.

Processing and quantification of the previously described imaging data employed multiple packages including MRtrix3 and the various libraries available within the ANTsX software ecosystem (Fig. 1). Derived imaging data used in this study included cortical thickness maps from T1-weighted MRI, diffusivity-based scalar images (e.g., fractional anisotropy) from diffusion-weighted MRI, functional scalar activation summarized via amplitude of low-frequency fluctuation (ALFF), and network correlation maps. Summary perfusion and PET scalar images were also generated for each subject. ANTs tools^27^ were used to register each T1-weighted image to an average population template as used in previous studies.^28^ Subsequently, each subject-specific scalar image was aligned to the corresponding T1-weighted image, thus providing a set of transformations to warp each image to the common template space.^27^

Details concerning modality-specific processing are as follows.

#### Diffusion-weighted imaging (DWI)

The standard MRtrix3 pipeline^29^ was used for diffusion tensor reconstruction and subsequent generation of diffusivity maps, specifically those corresponding to fractional anisotropy (FA) and radial diffusivity (RD). Pre-processing steps included denoising,^30^ whole-brain extraction,^31^ and tensor reconstruction.^32^ The average diffusion-weighted MR image was normalized to the corresponding T1-weighted MR image. Motion and eddy current correction were incorporated into the pipeline using the dwifslpreproc MRtrix3 program, which interfaces with the underlying FMRIB Software Library commands eddy, topup, and applytopup.^33,34^

#### Cortical thickness

ANTsX employs a registration-based framework for estimating cortical thickness^35^ that has been tailored and evaluated for both cross-sectional and longitudinal^36^ MR data. More recently, a deep-learning-based framework has been developed and evaluated^37^ demonstrating both superior measurement quality and increased computational efficiency. Briefly, processing includes brain extraction, brain parcellation based on six tissue types (i.e., cerebrospinal fluid [CSF], gray matter, white matter, deep gray matter, brainstem, and cerebellum), and application of the diffeomorphic registration based cortical thickness (DiReCT) cortical thickness algorithm.^35^ This yields a scalar image with non-zero values in the cortical gray matter providing a voxelwise estimate of thickness (in millimeters).

#### Resting-state fMRI

Resting-state functional MRI (fMRI) processing, performed in the R-based ANTs interface (ANTsR), has been described previously.^8,38^ Briefly, motion correction was applied to each time series. Nuisance parameters included the transformation parameters, the framewise displacement,^39^ and component-based method (CompCor) contributions.^40^ Summary images from the corrected time series included fractional amplitude of low-frequency fluctuation (fALFF).^41,42^

#### Cerebral blood flow (CBF) from first-pass perfusion imaging

Similar to the other image time series, the mean spatial image was generated and used as the reference image for motion correction. CBF images were generated using a singular value decomposition technique^43^ with the arterial input function generated automatically.^44^ Visual inspection of the CBF images revealed non-biological artifacts that persisted after standard confound modeling and processing. Consequently, CBF was excluded from the statistical analysis.

#### PET

PET images were also motion-corrected using ANTsX tools. The toolbox for partial volume correction (PVC) in PET (PETPVC),^45^ which provides several published methods for performing PVC, was used for additional pre-processing. Finally, after inspecting several anatomical regions and the range of PET activity, values were normalized for each subject based on the corresponding PET values in the left and right caudate. These segmented regions were identified using the corresponding T1-weighted image and the Desikan-Killiany-Tourville labeling protocol available in ANTsXNet.^37^

### Statistical analysis

The multifaceted neurological effects stemming from blast exposure necessitate multiple observations/measurements to characterize the associated structural, functional, and metabolic sequelae. In this study, the battery of measurements included multi-modal neuroimaging, self-reported or clinical neuropsychological assessment, and blood biomarkers. Such measurements can be inherently noisy and incomplete while simultaneously mutually overlapping and complementary. A recently developed statistical framework, known as similarity-driven multi-view linear reconstruction (SiMLR),^46^ has been employed for exploring and analyzing such data. Successful application includes a recent investigation into a career breacher cohort characterized by repetive low-level blast exposure^8^ where the use of SiMLR permitted identification of significant group effects spanning multiple modalities, including those mentioned previously.

Briefly, SiMLR is a multi-view extension of earlier techniques (i.e., single-view eigenanatomy^47,48^ and dual-view sparse canonical correlation analysis [SCCAN]).^49,50^ Each of these techniques is rooted in principal component analysis (PCA) with additional spatial constraints specifically tailored for neuroimaging data.^46^ Akin to PCA, these techniques are used for principled data dimensionality reduction—an important consideration in the context of neuroimaging where a single image can contain several orders of magnitude of statistically dependent data. In this way, and in contrast to standard mass voxelwise univariate^51^ or region of interest (ROI)-based approaches,^52,53^ statistical power is conserved by clustering data prior to statistical testing and adjustment for multiple comparisons, analogous to principal components regression.^54^

SiMLR's default setting identifies low-dimensional embeddings that optimize the joint predictive power between all modalities equally. We instead use a path modeling approach^46,55^ where SiMLR optimizes the predictive power from PET to the other modalities and from each MRI modality to PET. This strategy guarantees that the PET neuroinflammation modality drives the nature of the learned features across all modalities.

Formally, through SiMLR optimization, the $i^{th}$ modality, *X_i_*, is represented by a pair of matrices $\left(U_{i},V_{i}\right)$where the columns of *U_i_* are the low-dimensional bases spanning *X_i_* and the columns of *V_i_* are the regularized, sparse representation of each modality component. As mentioned previously, this permits principal component regressions of the form:

$$U_{i,l}∼d_{0}+d_{1}+\ldots +d_{n}$$

where the set of *d_j_* represents the explanatory variables (e.g., age, gender, and brain volume) and *l* denotes the specific basis vector of the $i^{th}$ solution.

To explore the relationship between GBEV, control, or exposed status (denoted as $arm_{t}$ below) and the imaging modalities, the following quasi-Poisson regression model was used:^56–58^

$$g\left(GBEV\right)∼\beta_{a}Age+\beta_{c}otherCon+\beta_{t}arm_{t}+\beta_{ml}U_{modality,l}$$

where each β is a coefficient, *g* is the *log* link function,^56^ *Age* is subject age, and *otherCon* represents the number of reported concussions from non-blast-related events. Quasi-Poisson regression models are appropriate for count or frequency data such as GBEV that is often non-Gaussian/overdispersed.^58^ The *modality* is one of *PET*, *CT* cortical thickness from T1-weighted MRI, *FA* (fractional anisotropy from DTI), *fALFF* (resting state), or *Jacobian* (a measure of localized volumetric change relative to a study template).^59^

### Critical GBEV threshold analysis

The above equation allows modeling the non-linear relationship between imaging variables and GBEV while controlling for baseline exposure level ($arm_{t}$), age, and concussions. The discrete control/exposed variable $arm_{t}$ defines a split of the cohort based on a GBEV threshold *t* where *t* varies between 9875.6 and 177,424 at discrete points determined by the values of our cohort. The threshold 65,309 corresponds to the original operator (*n* = 9) versus non-operator (*n* = 9) grouping. The regression parameter $\beta_{ml}$ on the term $U_{modality,l}$ is our key outcome of interest for statistical testing; it represents the degree to which the relationship between the modality's $l^{th}$ feature embedding and the total GBEV score accelerates in exposed subjects. In particular, we test for significant relationships between imaging variables (primary outcomes from PET with secondary outcomes from MRI) and GBEV after controlling for the other covariates. We assess significance of the $\beta_{ml}$ coefficients via permutation testing where the GBEV score is permuted 50,000 times per model. This empirical approach to *p*-value calculation is generally more conservative than model-based methods and leads to a minimum *p*-value of 2e-05.

### Analysis of biomarker and cognitive measurements

We adopt the same quasi-Poisson model as described above but instead use BDEVs or cognitive measures as predictors instead of SiMLR imaging components. In addition, we analyze these relationships only at a single threshold of ≥65,309, which is consistent with the original control versus exposed grouping.

## Results

Ten USSOCOM SOF SMs and 11 military control SMs were recruited into the current study. One subject from the exposed group was excluded due to demonstrating low-affinity binding TSPO genotype. One subject from the control group was also excluded based on a history of moderate to severe TBI revealed through the head injury questionnaire, the results of which met the criteria for prior severe TBI. An additional control subject withdrew prior to completing the study procedures. The final cohort included 9 controls and 9 exposed subjects with complete data. No significant difference was found between groups both in terms of age (Exposed: 41.9 years ±5.8 and Control: 41.6 years ±5.6, *p* = 0.9) or duration of service (Exposed: 21.3 years ±4.3, Control: 19.9 ± 6.6, *p* = 0.6). Subjects were recruited from multiple branches of the military (Exposed: Army = 6, Navy = 1, Air Force = 1, Marine Corps = 1; Control: Army = 4, Navy = 3, Air Force = 2) (Table 1). Blast-related concussion history differed between the two groups (Exposed: 1.7 ± 1.3, Control: 0 ± 0, *p* < 0.5). Additionally, non-blast-related concussion history differed between the two groups (Exposed: 3.9 ± 1.6, Control: 1.1 ± 1.4, *p* < 0.05). To control for non-blast-related concussion, this factor was included in the quasi-Poisson analysis, the results of which are outlined below.

**Table 1.** Demographic Summary of the Blast Exposed and Control Cohorts

	Exposed (*n* = 9)	Control (*n* = 9)
Age (years)	41.9 (range: 35–53)	41.6 (range: 36–51)
Handedness	Right = 7; left = 2	Right = 7; left = 1; ambidextrous = 1
Service	Army = 6	Army = 4
	Navy = 1	Navy = 3
	Air Force = 1	Air Force = 2
	Marine Corps = 1	
Duration of service (years)	21.3 (range: 8–27)	13.8 (range: 16–29)
Highest level of education	Doctorate = 1	Doctorate = 1
	Master's = 3	Master's = 5
	Bachelor's = 3	Bachelor's = 3
	Associate = 1	
	High School = 1	
GBEV	4,888,072 (112,393–16,534,930)	19,990 (0–65,309)

GBEV, generalized blast exposure value.

### Neuroimaging relationships to GBEV outcomes

**FIG. 2.**
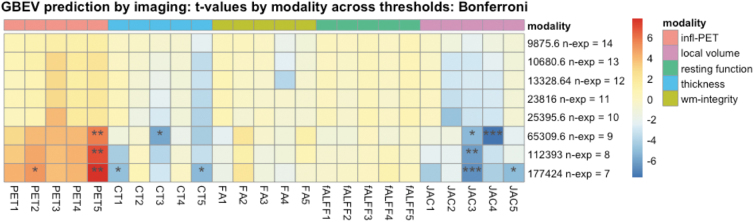
Threshold analysis for imaging predicting GBEV. This figure displays the *t-*statistic for each feature along with the permutation tested and Bonferroni-corrected significance level. At the 65,309 GBEV level (*n* = 9 exposed) we see an increase in the number of imaging measurements that significantly relate to exposure and these effects increase further at each successive level (CT3 and JAC4 excepted, see Discussion section). GBEV thresholds beyond 177,424 result in too few subjects for current models. Corrected significance denoted* for *p* < 0.05, *p* < 0.01, * for p < 0.005 and *** for *p* < 0.001. CT, cortical thickness; FA, fractional anisotropy; fALFF, fractional amplitude of low-frequency fluctuation; GBEV, generalized blast exposure value; JAC, Jacobian network; PET, positron emission tomography; WM, white matter.

**FIG. 3.**
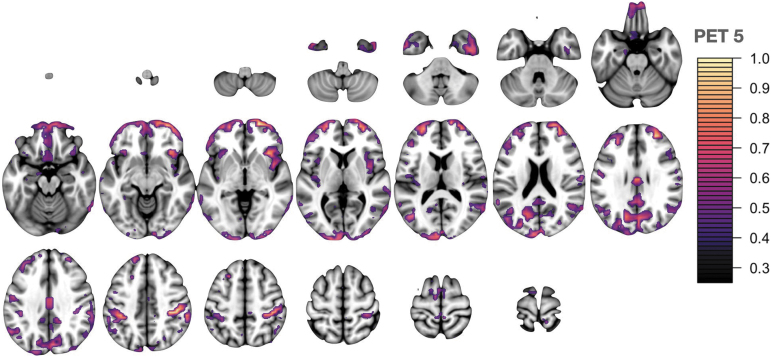
PET network 5 involves the salience network including the anterior insula and middle and superior frontal gyri. There is additional involvement of frontal pole and anterior temporal lobe. Signal is also contributed from precuneus, angular, and supramarginal gyri. Lastly, visual regions contribute including the cuneus and occipital pole. PET, positron emission tomography.

**FIG. 4.**
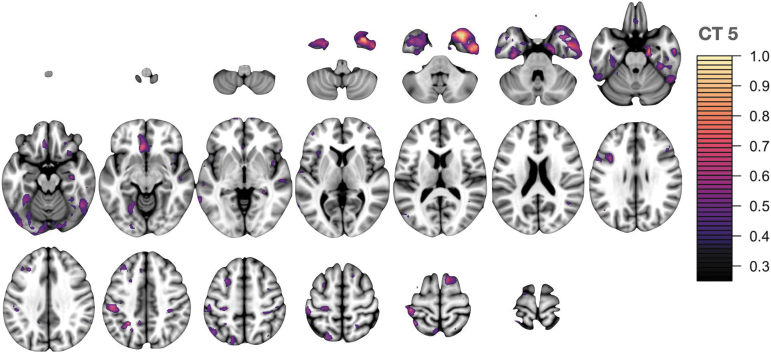
CT network 5. Primarily involves anterior temporal lobe regions including temporal pole, entorhinal cortex, and inferior temporal gyrus with additional involvement of the anterior cingulate, medial frontal cortex, and supramarginal gyrus. CT, cortical thickness.

**FIG. 5.**
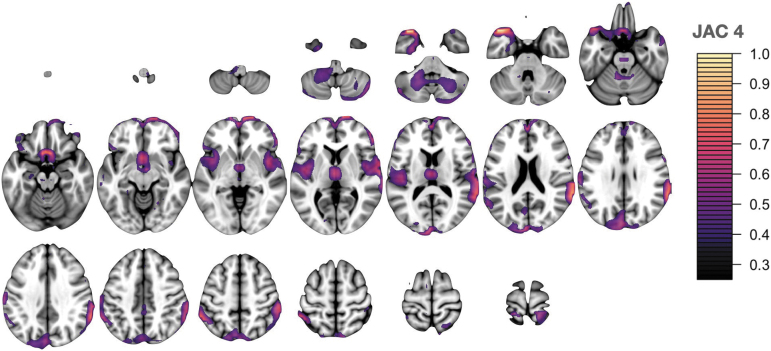
Jacobian/local volume network (JAC) 4 is dominated by cingulo-opercular task control network and ventral attention network regions including supramarginal gyri, temporal pole, and frontal pole.

**FIG. 6.**
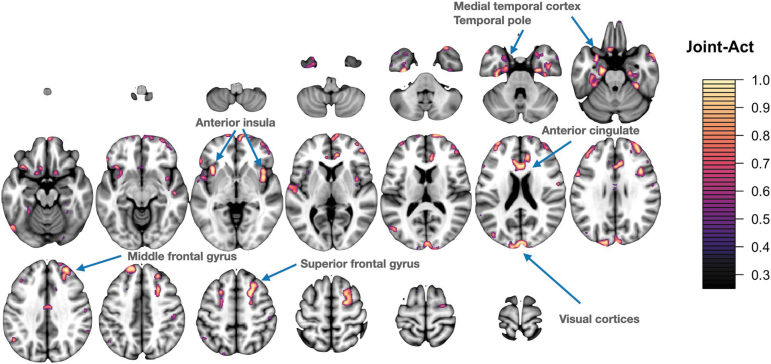
Spatially coincident features across all three modalities that reach Bonferroni significance. Neuroinflammation, reductions in thickness, and volume loss in these regions relate to increased GBEV scores. These cross-modality regions span the salience, task control, and default mode networks. GBEV, generalized blast exposure value.

**FIG. 7.**
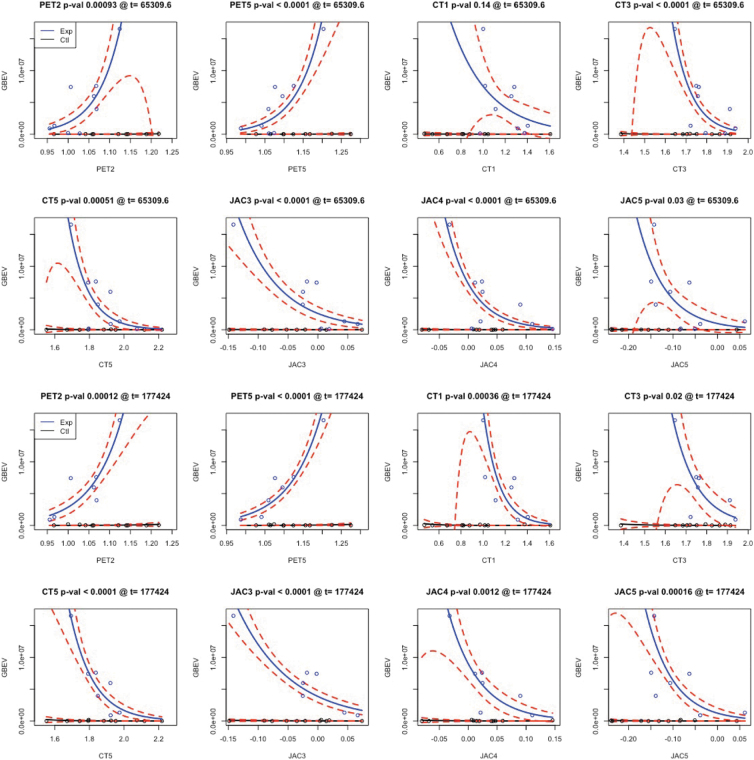
Neuroimaging relationships with GBEV score for both controls and exposed defined at two thresholds. Only predictors that survive Bonferroni correction at one of the two thresholds are shown. Dotted red lines indicate 95% confidence intervals around the best fit solid line. CT, cortical thickness; GBEV, generalized blast exposure value; JAC, Jacobian network; PET, positron emission tomography.

The critical threshold analysis involved 200 tests displaying the relationship between imaging and GBEV: five components for each of five modalities were tested across eight threshold levels. We corrected the *p*-values associated with each $\beta_{ml}$ using both Bonferroni (more conservative) and false discovery rate correction but focused on Bonferroni corrected results at the *p* < 0.05 level. The results of this analysis are summarized in Figure 2 with individual modality-specific features shown in Figure 3 for the most prominent PET feature, Figure 4 for the most prominent cortical thickness feature, and Figure 5 for the most prominent Jacobian feature. Note that in Figures 3–6, The values are the feature weights on the brain for a given component; regions with weights closer to 1 contribute more strongly to the derived weighted average (e.g., of neuroinflammation). Regions without color do not contribute at all. Overall, eight features survived this stringent *p*-value threshold spanning cortical thickness, neuroinflammation, and volumetric loss; these model relationships are shown in Figure 7. Recalling that *p*-values were computed with permutation tests, these surviving predictors should reflect the most reliable GBEV-associated imaging patterns within this cohort.

### Evidence for joint effects of exposure across modalities

SiMLR yields sparse patterns defining each feature vector. These patterns are unsigned weighted averages over the original voxelwise data matrix, that is, over the underlying neuroanatomy (Supplementary Fig. S1). Here, we demonstrate post hoc results that summarize the common anatomy involved in features derived from PET, cortical thickness, and volume loss. A visualization of the joint feature set is shown in Figure 6 as well as in Supplementary Figures S2–S5.

### BDEV and cognitive measurements

**FIG. 8.**
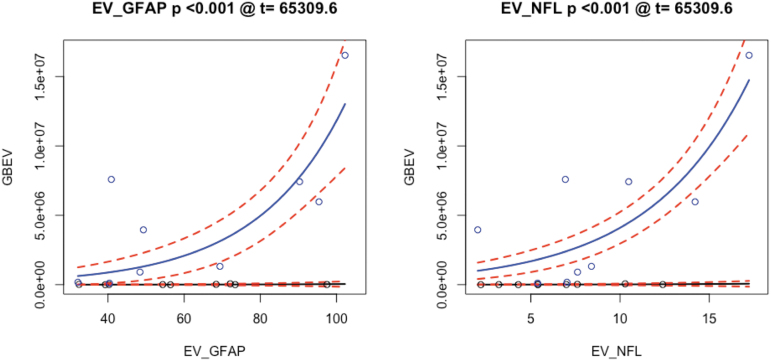
BDEV measures of neuroinflammation related to GBEV. BDEV, brain-derived extracellular vesicle; EV, extracellular vesicle; GBEV, generalized blast exposure value; GFAP, glial fibrillary acidic protein; NFL, neurofilament light chain.

The framework for analyzing cognitive and BDEV data is the same as that for imaging. However, we treat these predictors' relationship with GBEV independently, which results in a total of 23 tests: six BDEVs in addition to the NSI total score, the PGWI total score, the PCL-5 total score, the PSQI total score, and 12 measurements from ANAM 4 TBI-MIL. Of these, only two BDEV measurements survive Bonferroni correction. Figure 8 demonstrates the model relationships between GBEV and NfL and GFAP values.

## Discussion

The current investigation was performed in partnership with USSOCOM as a pilot study to explore the neurological sequelae of repeated low-level blast exposure in SOF SMs. Previous studies in other populations have demonstrated structural and functional alterations in repeatedly blast-exposed personnel such as breachers.^8^ As SOF SMs may be repeatedly exposed to blast overpressure during a career, it is important to better understand potential cumulative sequelae that may develop resulting from these exposures. The current study involved the acquisition of an array of measures, inclusive of self-reported information relevant to history of blast exposure, neuropsychological assessments, serum BDEV biomarkers, and neuroimaging inclusive of MRI and PET-CT. Based on previous evidence in repeatedly blast-exposed personnel, special emphasis was placed in the current study on an examination of the presence and extent of neuroinflammation through the visualization of an intravenously injected radioisotope targeting neuroinflammation with PET-CT as well as neuroinflammatory assessments within serum and BDEVs. These acquired measures generated a highly dimensional data set in a relatively small cohort.

The statistical model SiMLR was employed to identify low-dimensional embeddings that optimize the joint predictive power between all modalities equally and it is well-suited to handle the unique statistical challenges posed by such a data set. This model identifies specific networks or features (e.g., cortical thickness network 5 [CT5] or Jacobian network 4 [JAC4]) that are highly correlated with predictive measures of interest, such as blast exposure. This is the first work to show neuroimaging-derived critical GBEV thresholds for blast exposure.

The findings of the current study associate a history of blast exposure over a career with measures of increased neuroinflammation as well as alterations in brain structure in SOF SMs. Specifically, GBEV increases in exposed subjects as a function of both PET- and BDEV-related neuroinflammation measurements. Neuroinflammation levels, on their own, do not differ substantially between more highly and less highly exposed participants after controlling for potential confounds. However, within the exposed cohort, PET neuroinflammation measurements increase significantly with higher exposure levels. Additionally, MRI measurements demonstrate that increased blast exposure relates to reductions in volume and thickness of brain structures. These findings, together, establish that GBEV may provide an index into underlying effects of blast overpressure.

In addition to examining relationships between GBEV and neurological end-point assessments, a critical threshold analysis was performed to identify GBEV levels at which alterations in meaningful biomarker assessments may be observed. The GBEV critical threshold analysis reveals two types of effects: those that increase with threshold and those that are maximized within the exposed group membership level, that is, CT3 and JAC4. It is possible that results that are maximized only at the exact operator/control threshold may be a consequence of other operator-related effects in conjunction with neuroinflammation and/or blast exposure. If this is the case, it may suggest that the patterns captured by PET1, PET5, CT1, CT5, JAC3, and JAC5 are most specific to blast exposure effects. Of these, PET5 and JAC3 represent the statistically most robust features.

The neuroanatomical patterns revealed in this analysis may provide insight into potentially sensitive brain networks and/or mechanisms of injury. Both cerebellum and medial temporal structures are central to these patterns and these regions have been previously associated with blast injury in pre-clinical models^60–62^ inclusive of swine^63^ and non-human primates^64^ and in military SMs as well.^65,66^ The middle frontal and superior frontal gyrus (MFG, SFG) are also represented across modalities. Indeed, pattern FA4 shows large weights in the SFG at the FDR-corrected significance level, suggesting a more marginal, but still notable, relationship of GBEV with white matter integrity. Post-mortem studies of blast-exposed personnel have identified axonal injury within the MFG and SFG brain regions.^67^ Additionally, *in vivo* MRI has revealed blast-related gray matter volume loss in the SFG.^68^ These frontal regions play an important role in executive functioning, specifically working memory. The current work suggests that neuroinflammation may play a role in mediating structural alterations within these brain regions.

Limbic and temporal lobe regions are also implicated across three imaging modalities, inclusive of neuroinflammation, cortical thickness, and local volume. These findings are consistent with other studies involving examination of these brain regions, including a study of Iraq war veterans with repeated blast exposure and post-concussive symptoms, demonstrating cerebral hypometabolism in the medial temporal lobe.^65^ Also, veterans with blast-related TBI demonstrated magnetic resonance spectroscopy (MRS) evidence of decreased *N*-acetyl aspartate (NAA) to choline and NAA to creatine ratios within the hippocampus.^69^ Additionally, the hippocampus showed selective vulnerability to blast in an established mouse model.^70^ The consistent representation of anterior temporal lobe (ATL) in the results of the current study may be an indirect consequence of its network connectivity. ATL includes several densely connected regions critical to semantic memory and the hierarchical organization of category concepts.^71,72^ Disruption of these networks is associated with cognitive challenges of memory, language, and executive functioning.^73^

Volume reductions in cerebellum and brainstem are among the most heavily weighted structures within the local volume patterns and are linearly associated with GBEV scores. The cerebellum is connected to many of the regions that are prominent in our analysis of GBEV including medial temporal anatomy and, through the thalamus, to cingulate cortex and sensorimotor cortex. These cerebrocerebellar connections are important contributors to executive function and emotional regulation^74^ in addition to motor skills.^75^

It remains unclear whether cerebellar, temporal lobe, or frontal lobe injury occur concurrently or via a cascade of effects, or both. Future work is needed in larger cohorts to replicate and/or validate the sensitivity and/or precedence of blast effects on these circuitries. Although FA was used as the primary DTI-related outcome in this research, free water fraction may also be relevant to neuroinflammation^76^; this alternative approach may be pursued in future work.

We also cannot, in this cohort, disentangle specific effects of blast overpressure from those of concussion due to the high correlation between GBEV and blast-related concussion experiences. However, we partially mitigate this issue by controlling for concussions due to non-blast-related events. We also cannot rule out the contribution of behavioral or experiential confounds to the brain differences observed here.^67^ Such confounds may explain the relatively minimal difference in neuroinflammation between the two groups in the original arms of the study.

Cognitive measurements do not reveal direct relationships with GBEV. Larger sample sizes, different statistical modeling approaches, or alternative neuropsychological testing paradigms may be needed. NfL and GFAP BDEVs relate significantly (Bonferroni-corrected *p* < 0.05) to GBEV via quasi-Poisson modeling. NfL is relatively non-specific and this is reflected in its moderate association with concussion (uncorrected *p* < 0.001). GFAP has previously been associated with blast exposure, although a recent study indicated reduced levels with more acute exposure^77^—the opposite of the association found here.

SiMLR improves detection power by clustering neuroanatomical data into spatial patterns before hypothesis testing. The disadvantage of this procedure is that we do not directly test the roles of specific anatomy within the pattern. Rather, we infer the relative contribution by investigating the weight values. This type of interpretation must be approached with caution as the statistical tests only apply to the results of the whole pattern.

In summary, the current investigation demonstrated that increased blast exposure was associated with measures of increased neuroinflammation along with changes in brain structure in SOF SMs over a career. These observations survive Bonferroni multiple-comparison correction as well as control for potential confounds such as non-blast-related TBI. Additionally, critical threshold analyses demonstrate specific GBEV levels at which an association between increased blast exposure and alterations in neurological end-point assessments are observed.

The current investigation is a limited pilot study and caution should be exercised in interpretation of the findings of the study. Future hypothesis-driven research should be performed to determine the validity and overall reproducibility of these observed findings. Additionally, future studies with increased numbers of subjects should allow for a more complete critical threshold assessment. This final point is of importance in identifying and refining important dose-response relationships between blast overpressure exposure and potential injury metrics. Such thresholds may be of key importance in operational decision-making as well as the design of protective equipment to help mitigate the effects of blast exposure at the individual level.

## Transparency, Rigor, and Reproducibility Summary

The analysis plan was not formally pre-registered, but the lead author certifies that the analysis plan was pre-specified. The study was planned as an initial feasibility pilot study. As such, a formal power analysis was not performed at the outset of the investigation. Twenty-one subjects were recruited for the study, and data were successfully analyzed in 18 subjects. Participants were told the results of clinically significant or potentially clinically significant imaging findings after the final clinical observations had been made. Imaging acquisition and analyses were performed by team members blinded to relevant characteristics of the participants, and clinical outcomes were assessed by team members blinded to imaging results. MRI data were acquired using a Siemens Prisma 3T scanner, software version VE11C. All MRI data were acquired on the same scanner. All equipment and software used to perform imaging and pre-processing are widely available from commercial sources. The key inclusion criteria and outcome evaluations are established standards.

Neuroimaging processing and statistical analyses were performed by Brian B. Avants and Nicholas J. Tustison using the open-source ANTsR package, of which they are the founders and principal developers. Corrections for multiple comparisons were performed using false discovery rate available in the R statistical package. No replication or external validation studies have been performed or are planned/ongoing at this time to our knowledge. Analytic code used to conduct the analyses are available in (GitHub).

## Supplementary Material

Supplemental data

Supplemental data
