# Supplementary figures and images for: Neurological Effects of Repeated Blast Exposure in Special Operations Personnel
Source: J Neurotrauma. 2024 Apr 4;41(7-8):942–56. doi: 10.1089/neu.2023.0309 (PMC11001960; doi:10.1089/neu.2023.0309)

**Supplemental Figure 1:** Imaging relationships with other variables.


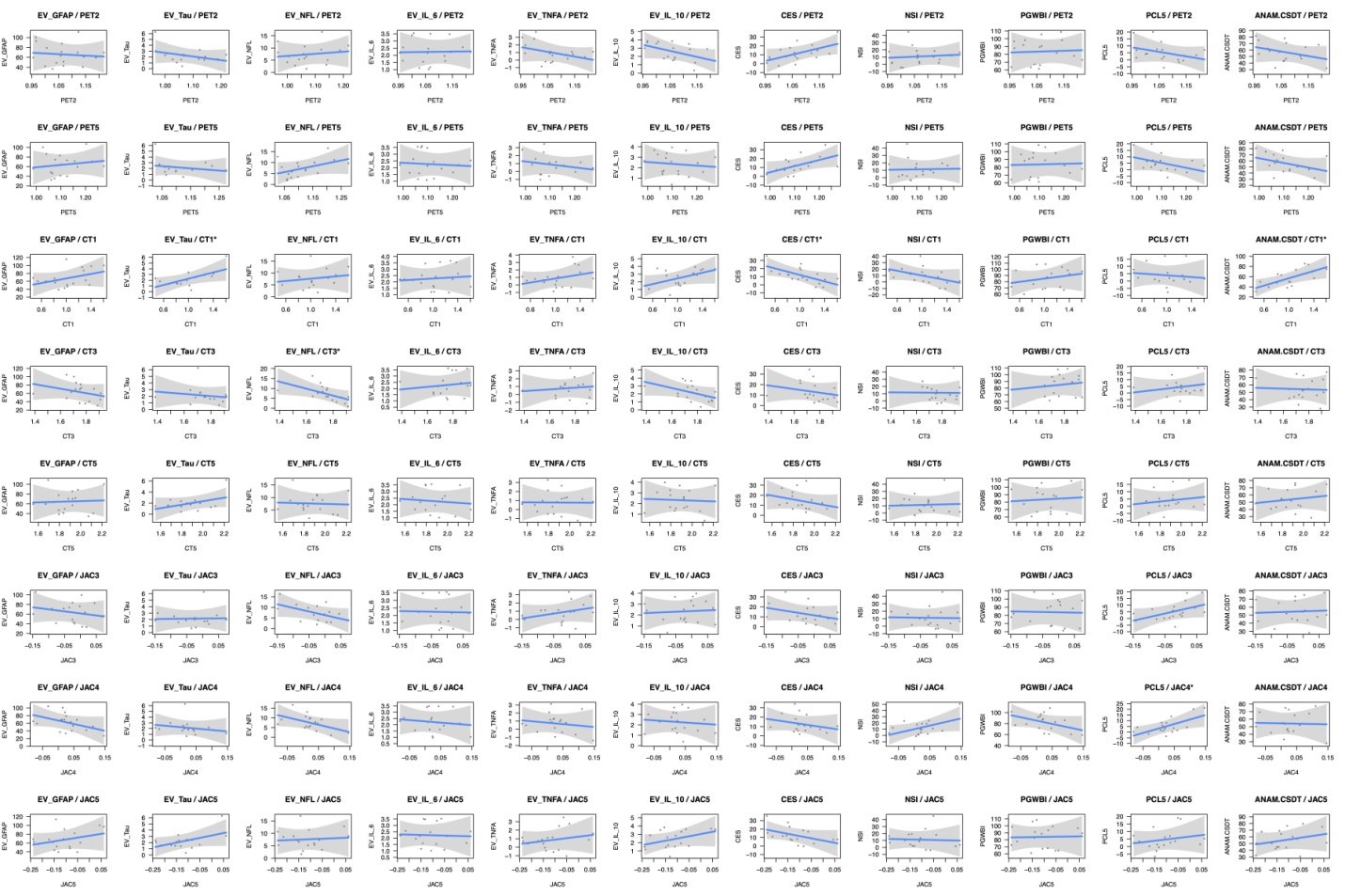

Supplement: Supplemental data [file Suppl_FigS1.docx]
