# Supplementary material for: Neurological Effects of Repeated Blast Exposure in Special Operations Personnel
Source: J Neurotrauma. 2024 Apr 4;41(7-8):942–56. doi: 10.1089/neu.2023.0309 (PMC11001960; doi:10.1089/neu.2023.0309)

**Supplemental Figure 2:** PET network 4 focuses in the inferior temporal lobe extending from anterior regions along medial and inferior cortical areas including the fusiform gyrus, parahippocampal, and entorhinal areas.


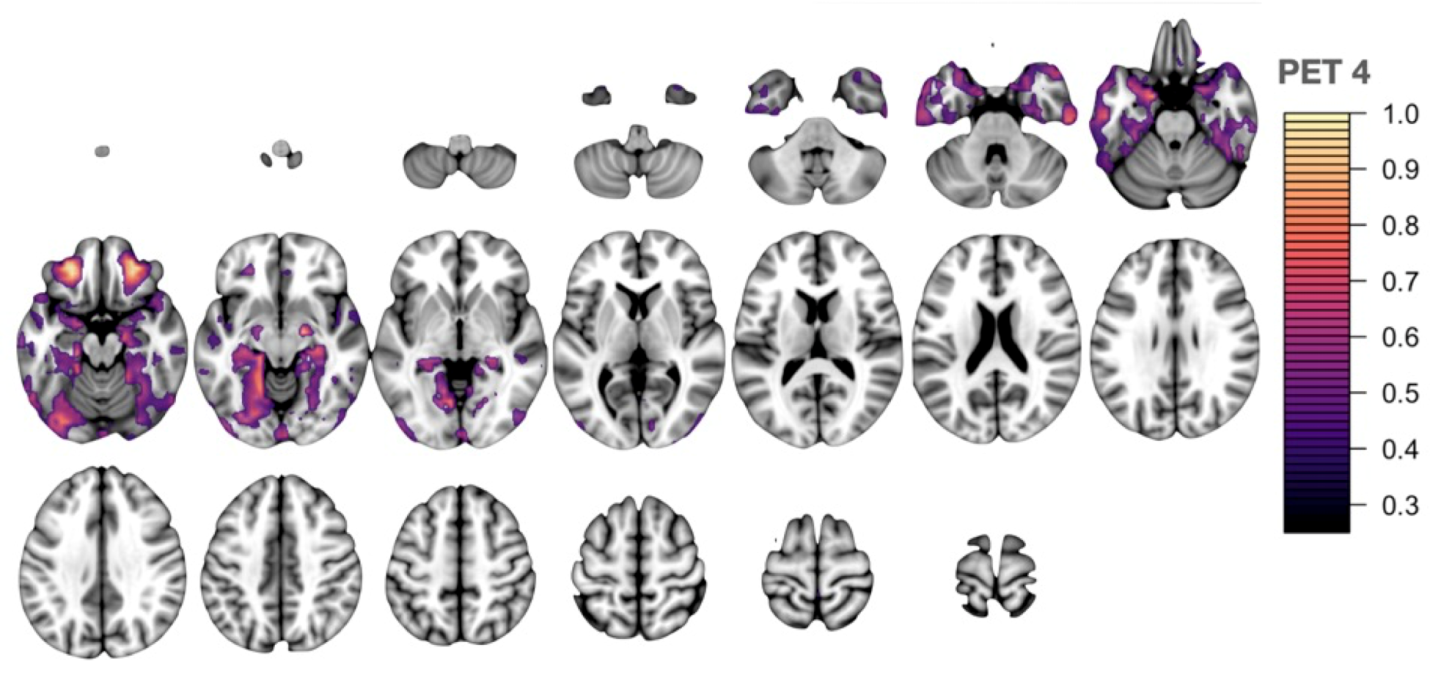


**Supplemental Figure 3:** Temporal lobe features span effects in PET neuroinflammation, local volume, and cortical thickness.


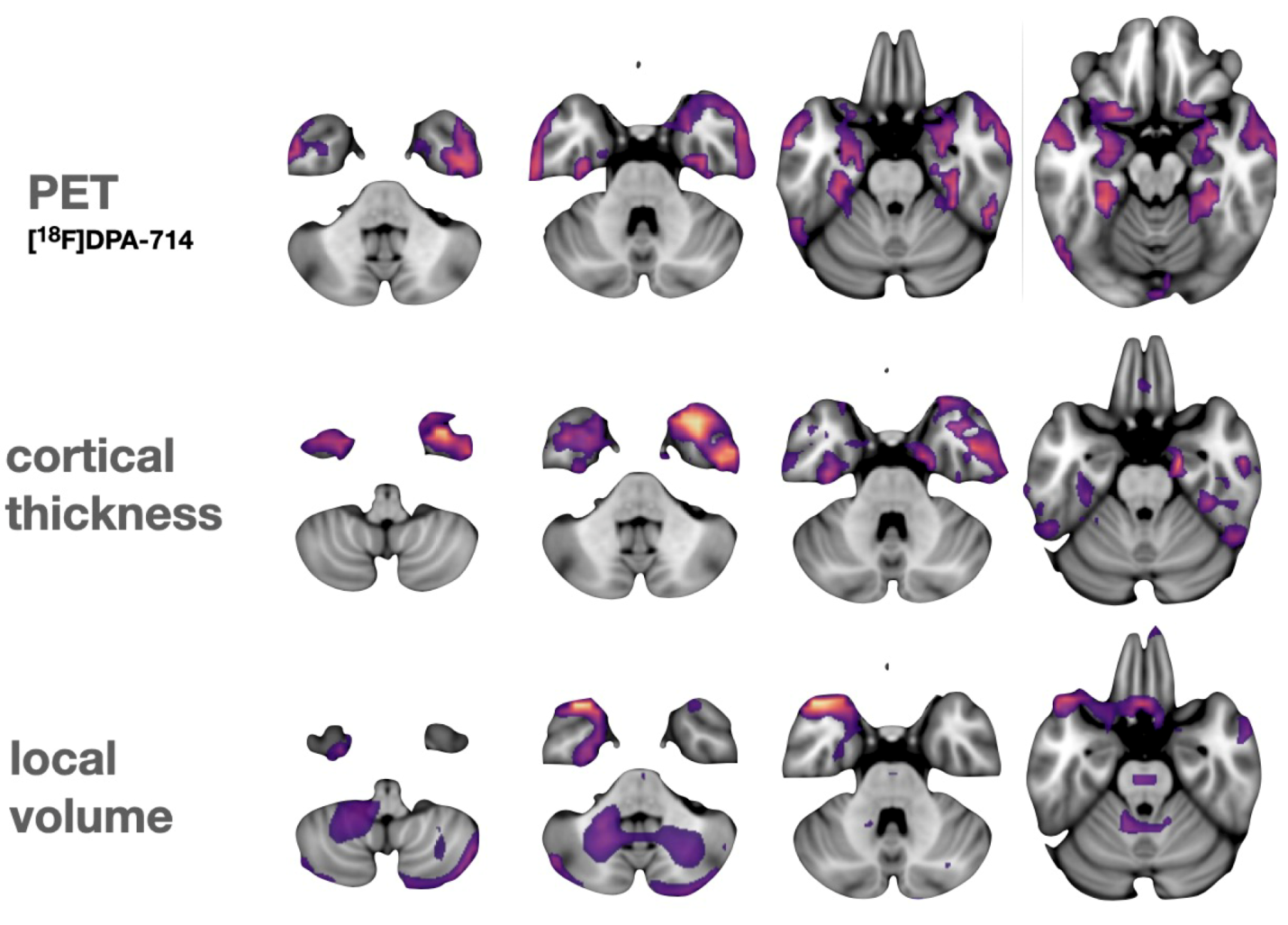


**Supplemental Figure 4:** Cerebellum features are among the most prominent volume loss components.


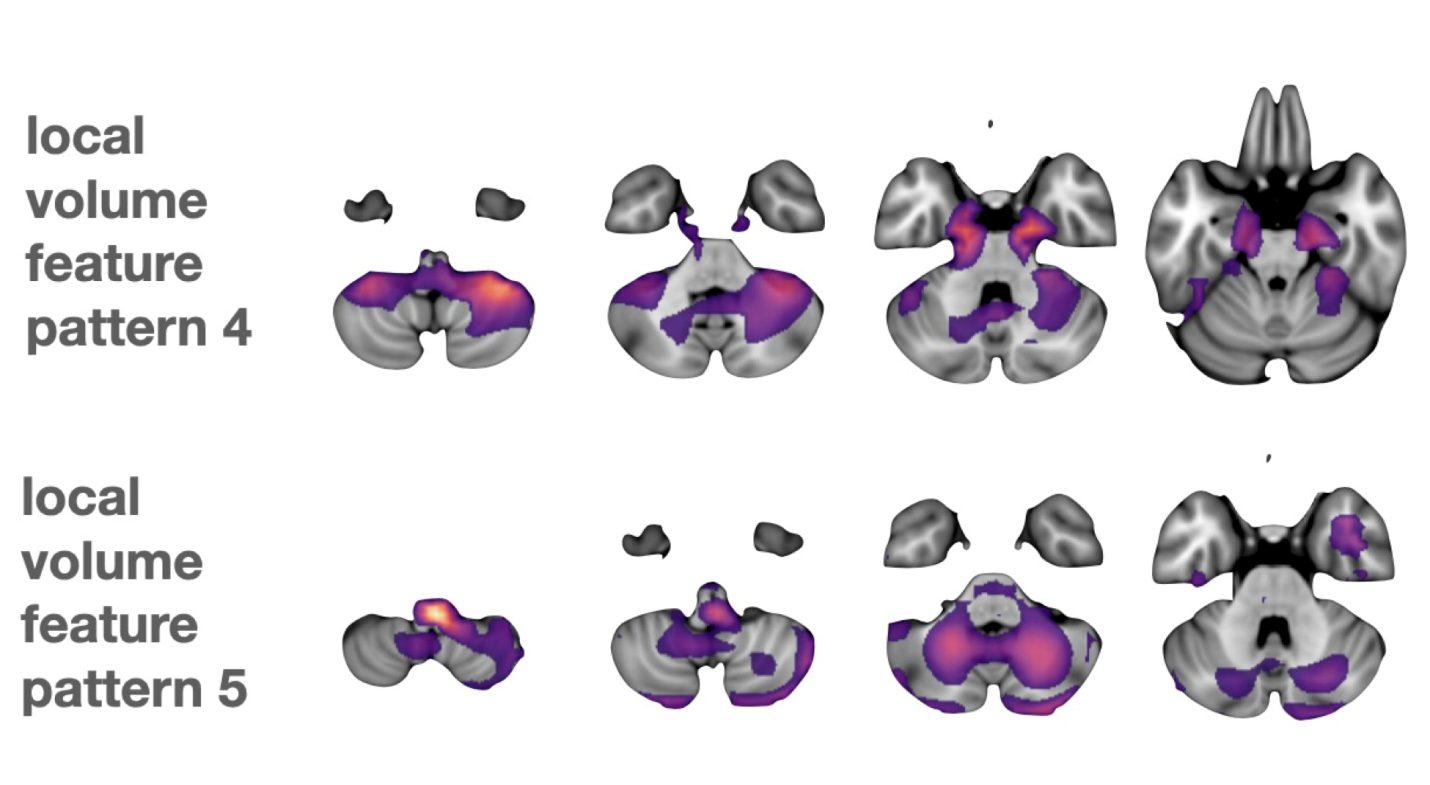


**Supplemental Figure 5:** Frontal gyrus effects across four modalities.


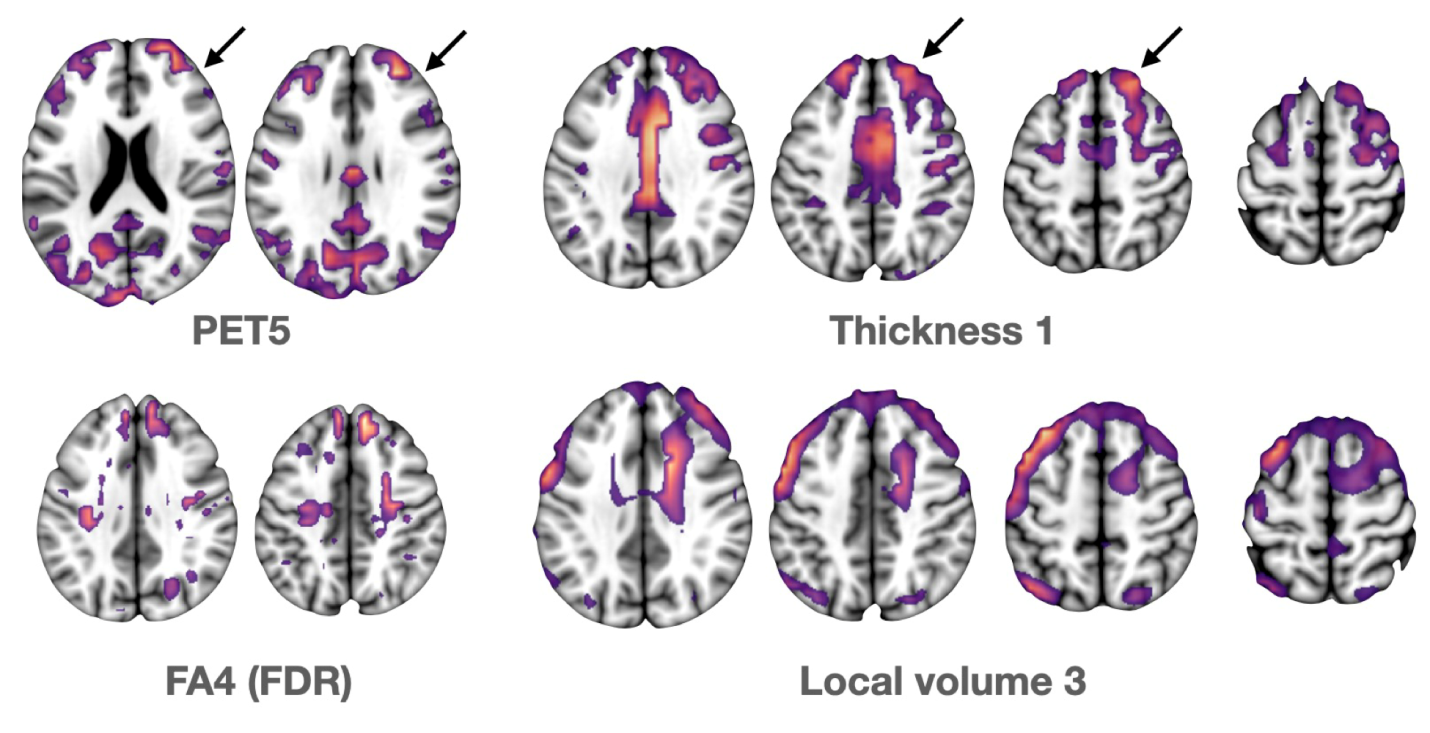

Supplement: Supplemental data [file Suppl_Fig_S2-S5.docx]
